# Ophthalmic manifestations of congenital protein C deficiency: a case report and mini review

**DOI:** 10.1186/s12886-020-01424-x

**Published:** 2020-07-13

**Authors:** Fariba Ghassemi, Fatemeh Abdi, Mandana Esfahani

**Affiliations:** 1 Eye research center, Farabi Eye Hospital, Tehran University of Medical Sciences, Qazvin Square, Tehran, IR Iran; 2 Retina & Vitreous Service, Farabi Eye Hospital, Tehran University of Medical Sciences, Tehran, IR Iran; 3 Department of Ophthalmology, Farabi Hospital Medical University Hospital, Tehran University of Medical Sciences, Tehran, IR Iran; 4 Department of Ophthalmology, Rasoul Akram Hospital, Iran University of Medical Sciences, Tehran, IR Iran

**Keywords:** Protein C deficiency, Congenital, Ophthalmic manifestations, Retinal detachment, Retinal dysplasia

## Abstract

**Background:**

Homozygous protein C (PC) deficiency is a potentially fatal disease with ocular blinding presentation or sequela.

**Case presentation:**

A 5 month-old boy was presented for evaluation of leukocoria. He had a history of frequent bruises and PC deficiency, treated with warfarin.

His intraocular pressure was normal. In the left eye leukoma with anterior segment dysgenesis, shallow anterior chamber, and cataract were observed. Fundus was not visible. B-scan revealed a closed funnel retinal detachment. His right eye had a normal anterior segment and a thin retina with anomalous retinal vascular branching at equator and peripheral retina. A fibrovascular tuft on the optic nerve head with induced traction on superior arcade was visible. Total loss of a and b wave of both were appreciated in electroretinography (ERG). Fluorescein angiography (FA) showed very severe leakage at the junction of the vascularized and non-vascularized retina and optic nerve head. Favorable outcome was achieved with lasering of avascular retina in the right eye.

**Conclusion:**

The potential for protein C deficiency should be assessed in all infants with leukocoria, anterior segment dysgenesis, retinal detachment and retinal dysplasia. Early diagnosis could save the child’s life and vision.

## Background

Protein C (PC), first described by Stenflo in 1976, is a vitamin K dependent anticoagulant enzyme that inactivates the plasma factors Va and VIIIa by limited proteolysis, thereby inhibiting the conversion of factor X to factor Xa and of prothrombin to thrombin [1]. Hereditary (congenital) PC deficiency is a rare autosomal disorder that predisposes to potentially blinding and fatal thromboembolic attacks [2]. Homozygotes have very low or undetectable PC activity (usually less than 1%, normal 70–140%) and do present within the first few days of life [2]. Heterozygotes have about 50% levels of PC and usually remain asymptomatic until adolescence or adulthood. Neonatal PC deficiency may also be acquired and transient, especially in preterm ill infants [3] with subsequent thrombosis being as severe as in the homozygous condition. Herein, we present a case of congenital PC deficiency with asymmetric ophthalmic manifestations. Fluorescein angiography (FA) and electroretinography (ERG) have been performed. The better eye was treated with indirect laser and the vision was saved.

## Case presentation

The patient was a full term Iranian male that was delivered with normal labor and was the first child of first cousin parents. He was presented on his third day of life with a purpura area on his buttocks and abdomen.

Very low protein C activity (less than 8%) was confirmed in the laboratory investigations and the patient was treated with fresh frozen plasma (FFP) and anticoagulant therapy (warfarin).

Both parents had low level of PC activity (52% in father and 60% in mother.). Liver and renal function tests were normal and no organomegaly or lymphadenopathy was observed. After 5 months, in his first ophthalmic examination, leukocoria was detected and evaluated. The child was on warfarin therapy with no more hypercoagulability state. The examination under anesthesia was conducted at Farabi Hospital for further assessment.

Both eyes had normal intraocular pressure (IOP). He had eye-to-eye contact in the right eye but he couldn’t fix and follow in left eyes.

**Fig. 1:**
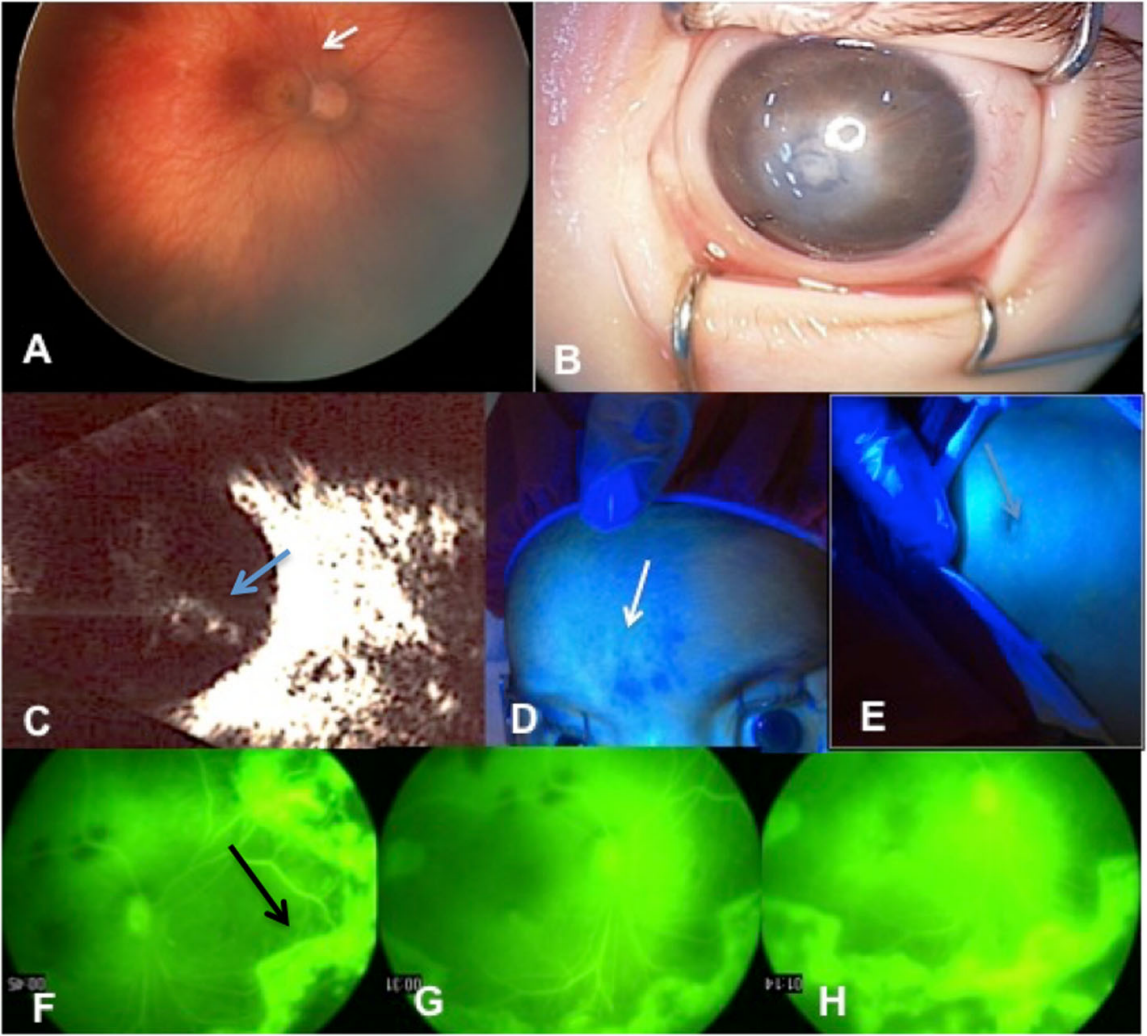
Ocular findings of a patient with homozygous congenital protein C deficiency. **a** In the right eye thin dysplastic retina and peripheral avascularization with a fibrovascular band from optic disc to superior arcade is visible (arrow). **b** In the left eye leukoma and irregular pupil with posterior synechia and dilated iris vessels and cataract is visible. **c** Total funnel shaped retinal detachment of left eye is visible in B-scan (arrow). **d** and **e** Splinter and patchy abnormal fluorescences of the skin at the time of fluorescein angiography are shown (arrow). **f**-**h** Fluorescein angiography with early leakage (arrow) from mid-peripheral vessels all over the retina and later leakage around the optic nerve head due to the tractional effect of fibrovascular band. The leakage increases during less than 1 min with severe vitreous fluorescence

His right eye had a normal anterior segment with round and reactive pupil. He had a central corneal leukoma on his left eye with a distorted pupil, total posterior synechia, dilated iris radial vessels, flat anterior chamber and mature membranous cataract. The right eye had a normal anterior segment, on a fundoscopy, a thin retina with anomalous retinal vascular branching and fading arterioles and venules was observed at equator and thereafter. (Fig. 1, a-h) There was a fibrovascular tuft on the optic nerve head with an induced traction on superior arcade. At the B-scan, the left eye had a closed funnel shaped retinal detachment. Electroretinography (ERG) elucidated complete loss of a and b wave of both eyes. Fluorescein angiography (FA) has shown very severe leakage at the vascularized and non-vascularized borders of the fundus and at the optic nerve head of the right eye. Severe patchy and stippled abnormal skin fluorescence (on the face, thorax, back, buttocks, and limbs) was observed at the time of FA. (Fig. 1, d-h) Indirect laser has been applied to the avascular areas.

The skipped areas were lasered during consecutive monthly follow-up visits and the neovascularizations were gone. The patient was followed-up for a total of 28 months with a favorable outcome, as no new ocular or systemic findings were detected. He had eye-to-eye contact and fix and follow vision in the right eye but no fix and follow vision in the left eye.

## Discussion and conclusions

Herein, we described a case of homozygote PC deficiency with severe asymmetric anterior and posterior segment dysgenesis. His right eye was rescued from the inevitable blindness of the future. The disadvantages of this report include the poor co-operation during ERG and FA (due to a young age) and the lack of genetic testing.

Activated PC prevents the formation of blood clots by down-regulating the coagulation cascade and blocking the spread of thrombosis [4]. Phenotypically, two types of PC deficiency have been described: type I, with reduced both antigenic and functional levels, and type II deficiency with reduced PC activity and normal antigenic concentration. Type I heterozygous cases comprise 76% of cases and homozygous type I comprise only 5% of cases with severe ocular and systemic findings. ^4^ Homozygous PC deficiency is rare with an estimated incidence of 1 in 500,000 to 1 in 750,000 [4]. It presents 2 h to months after birth, or with delayed symptoms, with life threatening thromboses involving the central nervous system, eyes, kidneys, and skin (purpura fulminans) [5]. Management is carried out in the acute phase with intravenous PC concentrate (ImmunoAG, Vienna, Austria), FFP and anticoagulators [5]. Untreated cases usually lead to death [5].

Ophthalmic manifestations include non-reactive pupils, leukocoria, chemosis, periorbital edema, shallow anterior chamber, dilated iris vessels, posterior synechia and microphthalmos. Posterior segment findings include vitreous, retinal and subretinal hemorrhage, retinal arterial and venous occlusion and retinal dysplasia [2].

One or both of the eyes may be affected. (Table 1) Leukocoria alone may be the first manifestation of homozygous PC deficiency, even before any other systemic manifestations such as purpura fulminans [17]. Early treatment may result in a better visual prognosis [17]. Likewise, homozygous PC deficiency can lead to thrombosis of the cavernous sinus and other cranial vessels [28]. These ocular complications may occur as antenatal or postnatal events [28].

**Table 1:** Review of the reported ocular problems in Congenital Protein C deficiency

Report	Reported cases	Involved eye	Anterior segment involvement	Posterior segment involvement	Global involvement
**Estelles A et al [1984]** [6]	1	OU		Bilateral vitreous hemorrhage and intravitreal mass	
**Marciniak E et al. [1985]** [7]	1	OU	Corneal opacity Case1: Corneal opacity, pupil not visible in one eye Case2: Bilateral cataract,	Case 1: Vitreous opacity in one eye Case 2: total retinal detachment with retinal new and old hemorrhages	Microphthalmia
**Rappaport ES et al. [1987]** [8]	1	OU		Hyperplastic vitreous bilaterally	
**Pulido JS [1987]** [9]	1	OU	Prominent iris vessels, shallow anterior chamber, synechia, cataract, retrolental membrane	Bilateral vitreal hemorrhage, and funnel-shaped retinal detachment in both eyes	
**Auletta MJ and Headington JT [1988]** [10]	1	OU	Raised intraocular pressure, flattened anterior chambers, iris atrophy, lens adhesions	Bilateral retinal detachment	Bilateral leukocoria
**Hartman KR [1989]** [11]	1	OU		Vitreal eye hemorrhages and	Intraparenchymal brain infarction
**Hermsen VM et al [1990]** [12]	1	OU	Vascularized lens, non-existent anterior chambers	Funnel-shaped retrolental mass, normal retina and optic nerve	Bilateral PHPV, microphthalmos
**Soria JM et al. [1985]** [13]	1			Persistence of primary vitreous	Microphthalmia, irregular globe
**Cassels-Brown A et al. [1994]** [14]	2	OU	Case 1: Posterior embryotoxon, shallow anterior chambers, ectropion uvea, posterior synechia Case 2: marked periorbital edema and hemorrhagic conjunctival chemosis	Case 1: subconjunctival hemorrhage, a right sided retinal arterial occlusion and bilateral florid retinal hemorrhages with swollen hemorrhagic optic discs secondary to retinal venous occlusions, vitreous hemorrhages retinal detachment in one eye Case 2: hazy media, minor bilateral vitreous hemorrhages, and minor right sided retinal hemorrhage	Case 1: Strabismus, nystagmus, leukocoria Case 2: early birth 25 wk., end up to death at 23 days of life because of some subarachnoid hemorrhage and with worsening lung function
**Acheson JF et al. [1994]** [15]	4	OD		Bilateral recurrent ischemic optic neuropathy	Association with Protein S deficiency, antithrombin III
**Dreyfus M et al. [1995]** [16]	9	OU			Unilateral or bilateral blindness 6/9 cases bilateral PHPV
**Hattenbach LO et al. [1999]** [5]	2	OU	Case 1. Shallow anterior chamber Case 2. Shallow anterior chamber, posterior synechia	Case 1. Left retrolental opacities, funnel retinal detachment, vitreous hemorrhage Case 2. Right vitreous hemorrhage, funnel retinal detachment	Case 1. Strabismus, microphthalmos Case 2. Microphthalmos
**Ergenekon E et al. [2000]** [17]	1	OS		Eye ultrasound revealed an10x7 mm hyperechogenic structure underneath. Retina of the left eye consistent with subretinal hemorrhage	Leukocoria
**Churchill AJ et al. [2001]** [18]	2	OU	Case2. Right leukocoria	Case 1. Bilateral central retinal vein occlusions, vitreous hemorrhage, and a right central retinal artery occlusion Case 2. Total retinal detachment and a left macular hemorrhage	
**Paysse EA [2002]** [19]	1	OU	Flat anterior chamber	Bilateral retinal detachment, fibrotic hyaloid arteries	
**Sirachainan N et al [2003]** [20]	2	OU			Preserving eye function, high myopia, cerebral palsy
**Park UC et al. [2005]** [21]	1	OU	Bilateral corneal opacity, microphthalmia, posterior synechia, pupillary membrane, shallow anterior chamber	Vitreoretinopathy suggesting, PHPV, intravitreal masses, funnel-shaped retinal detachment with bilateral retinal dysplasia	Bilateral leukocoria, no microphthalmos, blind OU
**de Lemus-Varela ML [2005)** [22]	1	OU		Vitreous hemorrhage	
**Douglas AG et al. [2010]** [23]	1	OU	No red reflex OU	Vitreoretinal dysplasia, severe hazy media in one eye and OU PFV	Leukocoria OS, bilateral microphthalmia,, deranged VEP, poor vision, nystagmus
**Wang BZ [2012]** [24]	1	OU		Macular hypoplasia with blond retinal background	Doll’s eye movements, no fix and follow movement of the eyes, ponto-cerebellar hypoplasia, nystagmus, strabismus, Flat ERG
**Desai S et.al. [2014]** [25]	1	OU		Combined central retinal venous and arterial obstruction	Severe Type II protein C deficiency with factor V Leiden mutation, Glaucoma
**Almarzouki HS et al [2016]** [26]	2	OU	Peter’s anomaly		
**Baothman AA et al. [2017]** [27]	5	OU	Peter’s anomaly, corneal opacity (3/5)	Blindness (4/5OU), ocular hemorrhage shortly after birth resulted in visual loss (1/5)	
**Present study**	1	OU	Left eye shallow anterior chamber, cataract, leukoma, posterior synechia	Dysplastic retina and total retinal detachment in the other eye	Flat ERG, fluorescent spots

*ERG* Electroretinography, *OD* Right eye, *OS* Left eye, *OU* Both eyes, *PHPV* Persistent primary hyperplastic vitreous

Systemic manifestations are purpura fulminans, hematomas; epistaxis; prolonged bleeding; hydrocephalus; subarachnoid hemorrhage; pulmonary embolism; thrombotic hemorrhagic gastrointestinal and genitourinary mucosal infarctions of renal and deep vein thrombosis with the pulmonary embolism and candida sepsis [28]. These lesions usually cause death if they are not treated.

Approximately 20 case reports of PC deficiencies with ocular involvement have been published since the first patient was described in 1981. (Table 1).

The casual association of PC deficiency and retinal dysplasia may be the case in our patient. A thrombosis of the fetal hyaloid arterial system and partial or complete microvascular occlusions at a crucial developmental stage may underlie the developmental eye pathology. Our patient had bilateral asymmetric involvement, and more specifically, avascularization and peripheral vascular leakage in the better eye and total funnel detachment in the other eye. This is the third bilateral asymmetric case reported and the only case with fluorescein angiography. The interesting problem in our case was the patchy severe fluorescence of some parts of the skin that was observed at the time of fluorescein angiography. Lasering of the avascular retina prevented the baby from being completely blind. Mosnier et al. documented the additional effect of protein C in on the cytoprotective pathway mediated by a separate mechanism than its anticoagulant role [29]. Maintaining the endothelial barrier and anti-apoptotic activity of the PC in this pathway [29] and vascularization arrest of retina may have some effects on retinal dysplastic changes. In some of the described patients, persistent primary hyperplastic vitreous (PHPV) and microphthalmia have been reported. (Table 1) Vitreoretinopathy in both eyes proposed retinal dysplasia rather than PHPV [21]. Depending on the time at which vascular insults occur within the ocular system during the embryonic developmental phase, different presentations of the anterior segment and /or posterior segment would follow. Despite complete treatment, the process of ocular changes could be progressive as seen in corneal opacity [21]. Wang et al. reported association of PC deficiency with other hypercoagulability states as protein S and antithrombin III deficiency, as well as borderline hypothyroidism [24]. We have not seen such an association.

We therefore conclude that unilateral or bilateral infantile ocular findings such as leukocoria and retinal detachments and dysplasia may be the first manifestations of homozygous PC deficiency. In addition, by paying attention to the possible asymmetric nature of the ocular manifestation, vision can be protected to some degree. Physicians should be aware of these problems, as these infants may also have severe cerebral and internal vital organ complications that are preventable, if they are treated on time. The possible effect of natural blood anticoagulants (such as PC) on the development of the anterior and posterior segments of the eye during or after fetal development has yet to be defined.

## Data Availability

All data generated or analyzed during this study are included in this published article.

## Abbreviations

ERG: Electroretinography
FFP: Fresh frozen plasma
PC: Protein C
PHPV: Persistent primary hyperplastic vitreous
